# Internal carotid artery aneurysm presenting as diplopia via telemedicine during COVID-19

**DOI:** 10.1177/1357633X20985392

**Published:** 2021-01-07

**Authors:** Sally L Baxter, David E Kuo, Shira L Robbins

**Affiliations:** 1 Viterbi Family Department of Ophthalmology and Shiley Eye Institute, University of California San Diego, La Jolla, CA, USA; 2 Health Department of Biomedical Informatics, University of California San Diego, La Jolla, CA, USA

**Keywords:** Remote consultation, tele-ophthalmology, telehealth, telemedicine, teleneurology, COVID-19, pandemic

## Abstract

A patient presented with acute onset of double vision during the start of the COVID-19 pandemic when elective medical care was restricted. Initially declining an in-person evaluation, she was examined using a telehealth video visit, incorporating multiple technological modalities to ascertain ophthalmic examination elements. Her findings prompted emergent neuroimaging, revealing a giant internal carotid artery aneurysm, which was successfully embolized to prevent debilitating and possibly fatal intracranial haemorrhage. This case report illustrates the successful use of telemedicine and remote patient data acquisition to make a life-saving diagnosis.

## Introduction

Double vision, or diplopia, can arise from a host of diseases ranging from benign to life-threatening.^1^ Broadly speaking, monocular diplopia (when there is double vision when only opening one eye) generally arises from benign conditions limited to the eye itself, such as refractive errors, cataracts, retinal disease, or even ocular surface issues such as dry eye. Binocular diplopia, when double vision occurs *only* with both eyes open (i.e. it disappears when one eye is covered), arises from eye misalignment. This can result from benign causes such as decompensated phorias, convergence insufficiency, or divergence insufficiency. However, binocular diplopia can also result from a more concerning range of causes, including neurogenic causes (i.e. cranial nerve palsies or myasthenia gravis) and orbital processes (such as thyroid eye diseases or tumours).^2^

Acute diplopia is a relatively common complaint in emergency settings, and prior retrospective studies have demonstrated a substantial proportion of patients (from 35% to over 80%) had diplopia secondary to an identifiable underlying cause.^3–5^ The initial diagnostic approach for diplopia generally consists of history-taking and a detailed neurological and ophthalmological examination, focusing especially on ocular motility and alignment.^1^,^6^

With widespread restrictions on in-person interactions in response to the COVID-19 pandemic in spring 2020, telemedicine and virtual care have witnessed an explosive increase in adoption.^7^,^8^ Massive reductions in face-to-face evaluations across all fields of medicine were seen due to local, state, and federal regulations and recommendations, medical society recommendations, and physician and patient preferences.

While many elements of an ophthalmological examination can be obtained via telemedicine,^9^ certain data points are difficult or impossible to obtain via video or phone interaction alone. A multitude of mobile apps have been used to fill this need, measuring exam elements such as visual acuity testing, colour testing, perimetry (visual field) testing, ocular motility recording, and fundus imaging.^10^,^11^ In this case report, we describe the evaluation of a patient presenting with acute diplopia during the COVID-19 pandemic using a combination of phone interactions, video visits, and at-home app-based testing, ultimately resulting in life-saving diagnosis and treatment.

## Case report

A 59-year-old woman called the University of California San Diego Shiley Eye Institute triage line complaining of diplopia. At the time, spring 2020, governor-mandated restrictions related to the COVID-19 pandemic had temporarily suspended elective medical care. Over the phone, the patient described a history of sudden onset of horizontal binocular double vision, which she noticed one day prior while driving. During the preceding two weeks, she also felt intermittent episodes of the “room spinning,” although she endorsed prior similar vertiginous episodes in the past that had self-resolved. Additionally, she reported a new frontal headache on the day of presentation. She was advised to present to the ophthalmology clinic emergently for an in-person, face-to-face evaluation. However, she declined due to concerns regarding COVID-19 but agreed to a same-day telemedicine video visit.

Before the video visit, she was provided with resources and links for tools to help obtain relevant ophthalmic data. For visual acuity assessment, she was sent a link to a Snellen visual acuity chart that could be printed for use at home (Safe Eyes America, Washington, DC, USA). For extraocular movements, she was asked to use a mobile application (9 Gaze app, See Vision LLC, Richmond, VA, USA) to document eye motility and alignment in the nine cardinal positions of gaze. The app provides a guide to users to facilitate correct eye positioning and image consistency and produces a composite image of the photographs for easy exporting.

The video visit was conducted with a strabismus specialist using a telemedicine platform (Doxy.me LLC, Rochester, NY, USA) compliant with the Health Insurance Portability and Accountability Act. The patient wore bifocals for distance and near-vision correction, but no other ocular history. Her medical history was notable for hypertension, which was well-controlled on losartan. She had no known medication allergies. She had undergone a prior Caesarean section but had no other surgical history. Her social history and family medical history was unremarkable.

**Figure 1.**
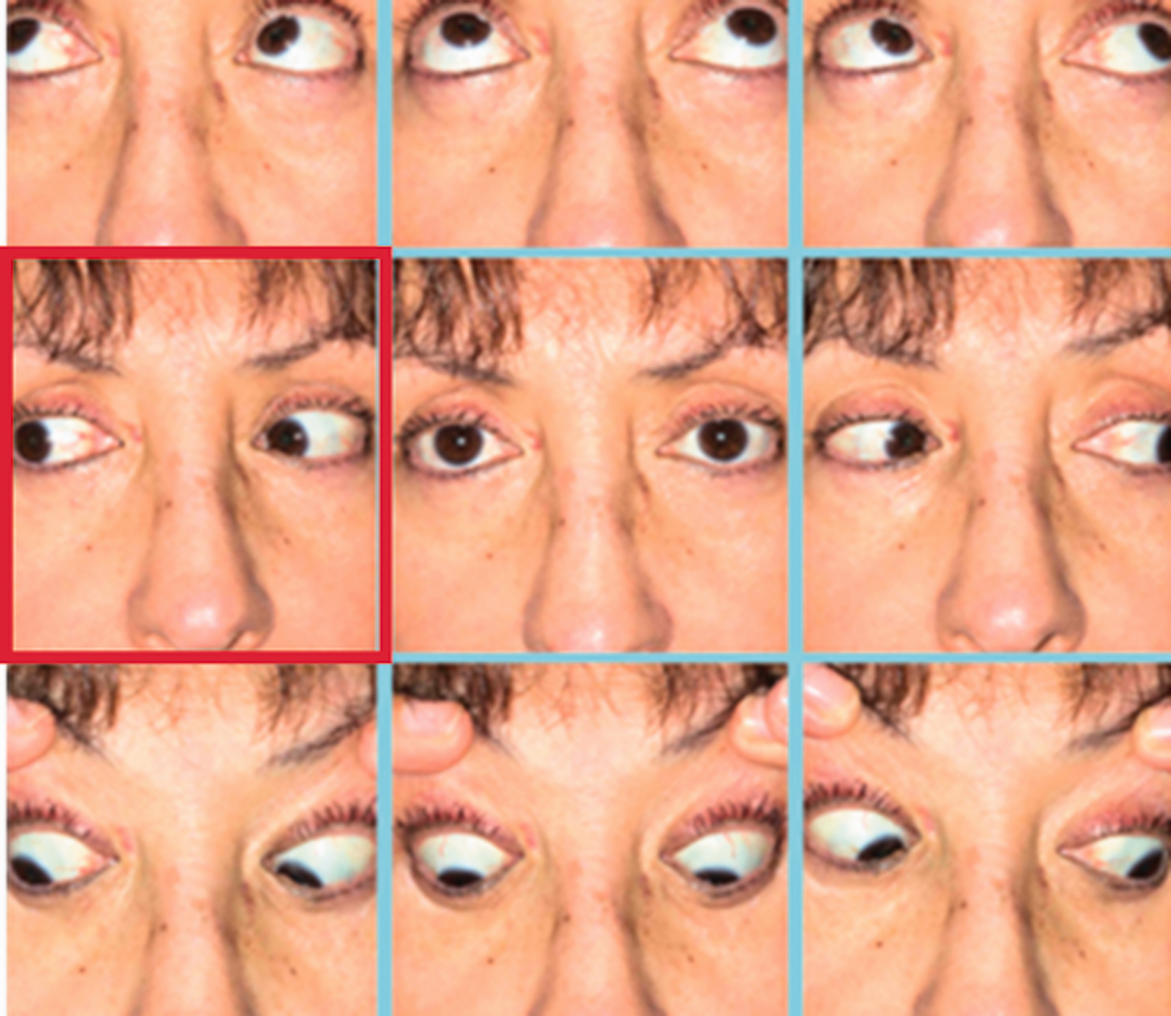
Photographic montage of eye positions for a patient presenting with acute binocular diplopia. The patient had acquired these images at home using a smartphone application (9 Gaze app, See Vision LLC, Richmond, VA, USA). She had a subtle abduction deficit in the right eye (middle image in the left-most column, highlighted in red), which was more noticeable during dynamic examination at the time of the telemedicine video visit.

Her visual acuity measured by the at-home Snellen chart was 20/20 in both eyes. Images from her motility examination obtained from the mobile app (Figure 1) were reviewed, and she was also asked to repeat the extraocular movements during the video visit. She was noted to have a subtle, small abduction deficit in the right eye, best seen when she turned her face to the left. In primary gaze, she had a small angle esotropia noted when she was verbally prompted to perform a cover–uncover test, with her own hand serving as an occluder. When prompted, the patient noted that the double vision was worse in right gaze. She could generate a normal saccade to abduction in the right eye. Taken together, these findings were consistent with an acute onset subtle sixth cranial nerve palsy. The remainder of her video examination, including pupil anatomy, confrontation visual fields, and gross examination of the ocular adnexa and anterior segment, appeared unremarkable.

**Figure 2.**
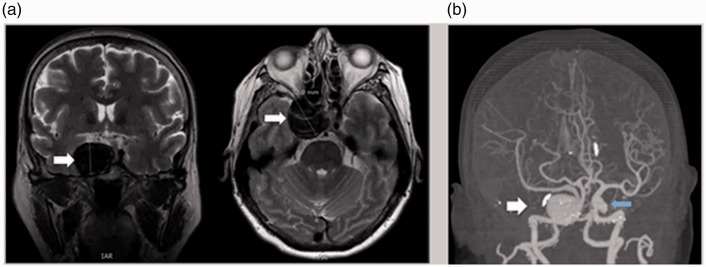
Giant internal carotid artery (ICA) aneurysm visualized on neuroimaging. The white arrows highlight various views of the partially thrombosed giant aneurysm arising from the right cavernous ICA, which measured 3.4 × 3.1 × 2.6 cm, on both magnetic resonance images (a) and computed tomography angiography (b). There was also a 2 mm aneurysm projecting posteriorly from the left ICA terminus (blue arrow in (b)).

A wide differential was considered, including tumour, ischemia/cerebrovascular accident, increased intracranial pressure, trauma, infection, inflammation, and migraine. Other possibilities included orbital mass, myasthenia gravis, and thyroid eye disease. Initial laboratory work-up included a complete blood count, basic metabolic panel, anti-acetylcholine receptor antibodies, and thyroid-related testing. Emergent neuroimaging was ordered, including computed tomography (CT) of the head and magnetic resonance imaging (MRI) of the brain and orbits with and without contrast. Findings on the MRI were concerning for an aneurysm, which prompted further investigation with CT angiography of the head and neck. Taken together, the neuroimaging revealed a partially thrombosed and partially calcified giant right cavernous internal carotid artery (ICA) aneurysm, measuring 3.4 × 3.1 × 2.6 cm, which was unruptured (Figure 2). There was decreased enhancement of the right ophthalmic artery compared to the left, and indication of erosion of the sphenoid bone. There were also other small aneurysms visualized, including a 2 mm aneurysm projecting from the left ICA terminus, and a 1–2 mm aneurysm at the origin of the right superior cerebellar artery.

Given these findings, the patient was emergently referred to neurosurgery for consultation. Due to the large size of the aneurysm and its dysmorphic features making it high risk for rupture, the patient was not considered a candidate for open surgical management. She was immediately referred to the interventional neuroradiology/endovascular neurosurgery service and subsequently underwent pipeline flow-diverting embolization and coil embolization of the right ICA aneurysm. Of note, besides the neuroimaging acquisition and the embolization procedure itself, all neurosurgical consultations and follow-up visits were also conducted via telemedicine video visits. During her most recent visit to ophthalmology, about two months after initial presentation, she was doing well and endorsed improved diplopia, and her vertigo had resolved. She did not have any neurological or cognitive deficits.

This patient provided written consent for her history, exam, photographs, and imaging studies to be included in this report.

## Discussion

This report describes a middle-aged woman presenting with acute horizontal binocular diplopia due to a subtle sixth nerve palsy, which was identified via telemedicine evaluation. Her imaging revealed a giant ICA aneurysm, for which she underwent successful embolization. Her timely diagnosis avoided intracranial haemorrhage that could have been life-threatening or resulted in severe neurological deficits. Compressive intracranial aneurysms are a well-documented cause of ocular motor nerve palsies (which include the third, fourth, and sixth cranial nerves).^1^ These entities should be considered in the differential for acute binocular diplopia.

A dynamic physical exam offers immense value in the diagnosis of cranial nerve palsies and in the evaluation of diplopia more generally. In the office, the examiner and the patient’s head can be moved, facilitating assessment of ocular alignment in various gaze positions, head tilt positions, and at distance and at near. In contrast, in a telemedicine video visit, the patient defaults to looking straight ahead, and the view is two-dimensional. This patient’s cranial nerve palsy would not have been identified without a dynamic exam involving her turning her face and performing a cover–uncover test on herself. Despite having a giant aneurysm, her clinical presentation was subtle. In a telemedicine assessment of diplopia, verbally instructing patients to move their heads is critical to assess the full range of eye movements. More generally, physicians and other care providers should be cognizant of how to adapt their typical physical exam manoeuvres in the office for virtual formats, with deliberate consideration of verbal and/or visual instructions for patients.

This patient’s story illustrates some of the opportunities presented by telemedicine and its growing prevalence. Thanks to the availability of telemedicine tools and infrastructure to conduct the initial evaluation virtually, this patient was quickly identified as requiring emergent diagnostic work-up and treatment even without a face-to-face evaluation, which she initially declined due to concerns about COVID-19. Without the telemedicine evaluation leading to the diagnosis and treatment of her aneurysm, she likely would have suffered serious adverse consequences. Similarly, other studies have also demonstrated that telemedicine visits during COVID-19 have facilitated identification of emergent conditions and specifically neurological emergencies.^12^,^13^

However, telemedicine also presents many challenges. First, despite the widespread adoption of telemedicine, the US Centers for Disease Control and Prevention estimates that as of June 2020, 41% of US adults delayed or avoided medical care during the pandemic, including 12% who avoided urgent or emergency care.^14^ These delays in care may increase morbidity or mortality. In addition, while telemedicine is effective for history-taking and some elements of the physical exam, there are still significant limitations to the virtual physical exam. One strategy has been to develop technologies for remote patient data collection, such as wearables and monitors that can measure vital signs at home. Other approaches include smartphone apps and patient portals connected to electronic health records (EHRs). However, increasing dependence on these tools may serve to widen the “digital divide,” as patients have wide variations in health literacy, technology literacy, and access to these tools.^15^ While some have posited that telemedicine will improve access to care, in some respects the differential access to digital health technologies may serve to exacerbate existing disparities in healthcare. Finally, the expansion of telemedicine has blurred the lines between work and home for many providers, potentially worsening burnout. The burden of EHR “in basket” messages was already high prior to the pandemic,^16^ and the transition to telemedicine has only increased the burden of clinicians being available 24/7 for patient needs.^17^ As physician burnout was already reaching epidemic levels prior to the COVID-19 crisis,^18^ understanding how to manage the additional burdens imposed by telemedicine will be critically important.

Despite the challenges, telemedicine is here to stay, and will transition from an emergency response strategy to becoming a routine component of healthcare delivery.^19^,^20^ This transition will require ongoing investments in training personnel on best practices for telemedicine encounters, developing long-term technology infrastructure for telemedicine, supporting sustainable funding mechanisms, and integrating telemedicine into clinical workflows.^20^ This is especially important for specialties and sub-specialties where use of telemedicine has been historically less robust than for primary care. For example, for specialty areas such as ophthalmology and neurology, telemedicine offers additional opportunities for expanding access to care in underserved and rural areas where specialists may not be easily accessible in person. Particularly for emergent consultations highlighted by this patient, telemedicine could end up being a life-saving intervention.
